# Cardioprotective effects of curcumin and piperine against obesity induced oxidative stress in rats

**DOI:** 10.1038/s41598-026-40407-8

**Published:** 2026-03-18

**Authors:** Hanan M. Rashwan, Eman O. Mohamed, N. M. M. Emam, Aida A. Hussein

**Affiliations:** 1 Zoology and Entomology Department, Faculty of Science, Arish University, North Sinai, Egypt; 2 Zoology Department, Faculty of Science, Suez University, Suez, Egypt

**Keywords:** Obesity, Cardiac biomarkers, Curcumin, Piperine, Oxidative stress, Inflammatory markers, Cell biology, Drug discovery, Immunology, Physiology, Plant sciences, Diseases

## Abstract

One of the adverse effects of obesity is cardiovascular disease (CVD). This study aimed to estimate the preventive effect of curcumin (CUR) (100 mg/kg body w.) and piperine (PIP) (5 mg/kg body weight) orally treated for 8 weeks in obese rats against CVD. When rats were fed a high-fat diet (HFD), high-density lipoprotein cholesterol (HDL-C), serum total cholesterol (TC), triglyceride (TG), Castellis risk index, CKD-II, atherogenic index (AC), and serum insulin increased. Results showed decreased levels of antioxidants catalase (CAT), glutathione peroxidase (Gpx), and superoxide dismutase (SOD), and increased levels of oxidative stress, including malondialdehyde (MDA), nitric oxide (NO), and reactive oxygen species (ROS), in the hearts of HFD rats. Other changes included a significant decrease in the activity of cardiac Na+/K+ ATPase, as well as increases in cardiac inflammatory markers, including TNF-α, IL-1, and IL-6. This was accompanied by further increases in serum C-reactive protein (CRP), monocyte chemotactic protein 1 (MCP1), and plasminogen activator inhibitor 1 (PAI1), as well as decreases in adiponectin levels. Furthermore, serum troponin T, creatine kinase-MB (CK-MB), and myoglobin levels were increased and downregulating NF-κB gene expression. Histological examination revealed multiple changes in the hearts of obese rats. CUR + PIP intake suppressed obesity-induced cardiac oxidative stress, inflammation, and histological damage. Thus, these results suggest that CUR + PIP intake may help in preventing obesity-related health risks and cardiac toxicity.

## Introduction

High-fat diets (HFDs) have become increasingly prevalent and are a major contributor to obesity, which the WHO defines as “excessive accumulation of fat in the body^1–3^. Obesity is strongly associated with metabolic disorders such as cardiovascular disease, diabetes, dyslipidemia, metabolic syndrome, and certain cancers⁴, highlighting the need for effective preventive strategies. Naturally derived compounds, particularly polyphenols, have gained attention for their pharmacological potential in managing oxidative stress-related diseases⁵. Curcumin (CUR), the main bioactive component in turmeric, exhibits antioxidant, anti-inflammatory, antimicrobial, and cardioprotective properties^6–8^. Piperine (PIP), a bioactive alkaloid from Piperaceae plants, not only has antioxidant and anti-inflammatory effects but also enhances the bioavailability of CUR and other drugs^9–13^. Despite numerous studies on CUR and PIP, most have focused on isolated biochemical endpoints or single-tissue analyses. Previous reports demonstrated that curcumin could modulate NF-κB signaling and calcium homeostasis in different disease models^14,15^, yet no research has comprehensively investigated these molecular and ionic mechanisms in obesity-induced cardiac dysfunction. Therefore, the present study aims to fill this knowledge gap by evaluating the cardioprotective potential of CUR + PIP in HFD-fed male rats. Parameters measured include lipid profile, insulin, oxidative stress markers, inflammatory cytokines, cardiac enzymes, ionic balance (Na⁺, Ca²⁺), NF-κB expression, and histopathological alterations. By providing this multi-level assessment, our work seeks to clarify how CUR + PIP mitigate obesity-related cardiac dysfunction and to offer a more integrated mechanistic perspective compared to existing literature.

## Materials & methods

### Chemicals

CUR + PIP was purchased from Puritan’s Pride, INC. DMSO was obtained from El-Gomhoria Company for Chemicals, Mansoura, Egypt.

### Animals & studied groups

Thirty adults male Wistar albino rats (weight: 180–200 g) were acquired from the Egyptian Institute of Serology and Vaccine Production (Helwan, Egypt) for this study. Rats were housed in stainless steel cages in an animal house with proper ventilation and standardized environmental control (23 ± 2 °C, 12-h light-dark cycle, and 40 ± 5% humidity). Food and water were available ad libitum at all times. After acclimatizing to the new environment for 1 week, rats were randomly divided into 5 groups (6 rats per group). Group I rats were fed a standard diet containing 8% fat, 75% carbohydrate, and 17% protein as a control. Group II rats were orally administered 5% DMSO via stomach tube at a dose of 0.1 mL/100 g body weight as a vehicle. Group III rats were orally administered Cur (100 mg/kg body weight) + Pip (5 mg/kg body weight) dissolved in 5% DMSO. Group IV was fed a HFD diet (60% fat, 23% carbohydrate, 17% protein)^16^ and group V was fed a HFD diet and further administered CUR + PIP orally in the manner and doses described for the above groups. Animals were monitored for body weight changes on a weekly basis throughout the 8-week study period and immediately before sacrifice.

### Ethical approval

All experimental protocols were approved by the Ethics Committee for Animal Research of the Faculty of Science, Arish University (approval number: ARU010). All methods were carried out in accordance with relevant guidelines and regulations. All methods are reported in accordance with ARRIVE guidelines (https://arriveguidelines.org).

### Samples collection

At the end of the trial period (8 weeks), all animals were fasted overnight and sacrificed under anesthesia (ketamine/xylazine, 0.1 ml/100 g b.w, intraperitoneally. Euthanasia was performed by a ventral neck incision using a sterile scalpel to sever the major cervical blood vessels, and blood was collected by free flow from the incision (rapid exsanguination) into labeled tubes^17^. Two samples of blood were taken from each rat; the first sample was collected without an anticoagulant, and the second sample was collected in sodium citrate tubes. Each sample was prepared and processed immediately after blood collection. The serum was centrifuged at 850 × g for 15 min and stored at -20 °C in labeled Eppendorf tubes for further biochemical tests. Rats were dissected, and hearts were isolated and washed with saline. The left side of the heart was homogenized in phosphate-buffered saline (PBS, 0.1 M, pH 7.4) (1/10 w/v) and centrifuged at 850 × g for 15 min. The supernatant was transferred to a labeled tube and stored at -20 °C until used for various biochemical tests. The right side of the heart was fixed in 20% neutral formalin for histological examination.

### Biochemical analysis

#### Lipid profile

In rat serum, total cholesterol (TC), triglycerides (TG), high-density lipoprotein cholesterol (HDL-C), and low-density lipoprotein cholesterol (LDL-C) were measured using kits from Bio-diagnostic, Egypt, as characterized by the methods of Allain et al.^18^, Fossati and Prencipe^19^, Lopes-Virella et al.^20^, and Rasouli and Mokhtari^21^, respectively.

#### Castelli’s risk index I (CRI-I), castelli’s risk index II (CRI-II), and atherogenic coefficient (AC)

Cardiovascular risk indices: CRI-I, CRI-II, and AC were calculated using the following formulas described by Lumu et al.^22^:

- CRI-I = TC/HDL.
- CRI-II = LDL/HDL.
- AC = (TC-HDL)/HDL.

### Serum insulin

Insulin concentrations were measured using a rat-specific ELISA kit (ALPCO, USA) according to the method of Findlay and Dillard^23^.

### Antioxidant and oxidative stress markers

In the heart homogenates, the levels of glutathione peroxidase (GPx), superoxide dismutase (SOD), and catalase (CAT) were assessed using kits obtained from Bio-diagnostic Co. (Dokki, Giza, Egypt) according to the methods of Paglia and Valentine^24^, Nishikimi et al.^25^, and Aebi^26^, respectively. Meanwhile, malondialdehyde (MDA) and nitric oxide (NO) concentrations were measured using kits from Bio-diagnostic Co. (Dokki, Giza, Egypt) according to the methods of Ohkawa et al.^27^ and Montgomery Dymock^28^, respectively. Reactive oxygen species (ROS) concentrations were assessed using an ELISA kit purchased from AFG Bioscience (Skokie Boulevard, Northbrook, USA) according to the manufacturer’s protocol. Approximately 0.1 g of heart tissue was homogenized in 1 ml of buffer, and the results obtained from heart homogenates (antioxidant enzymes, oxidative stress markers, inflammatory markers, Na⁺/K⁺-ATPase, and MCP-1) were expressed per g tissue.

### Na⁺/K⁺-ATPase activity

The activity of Na⁺/K⁺-ATPase was assessed in heart homogenates using an ELISA kit (MyBiosource, USA) according to the manufacturer’s protocol.

### Inflammatory markers

The levels of tumor necrosis factor (TNF-α), interleukin-1 (IL-1), and interleukin-6 (IL-6) in cardiac homogenates were measured using rat ELISA kits (Cloud-Clone Corp., USA) as described by Aukrust et al.,^29^ Grassi et al.,^30^, and Wang et al.,^31^, respectively.

### Determination of C-reactive protein (CRP), monocyte chemoattractant protein-1 (MCP-1), plasma plasminogen activator inhibitor-1 (PAI-1), and adiponectin levels

C-reactive protein levels in rat serum, as defined by Pepys^32^, were measured using a quantitative C-reactive protein kit from Sigma Diagnostics (EU). Monocyte chemotactic protein-1 concentrations in heart homogenates were estimated using a commercial ELISA kit (Cusabio Biotech, Houston, USA) according to the manufacturer’s protocol. Plasminogen activator inhibitor-1 concentrations in plasma were measured using a commercial kit from Cusabio (China) according to the manufacturer’s protocol. Adiponectin concentrations in serum were estimated using an enzyme-linked immunosorbent assay kit (Cusabio Biotech, Houston, USA) according to the manufacturer’s protocol.

### Cardiac biomarkers

#### Determination of troponin T

Serum troponin T levels were quantitatively measured using a rat troponin T ELISA kit from Kamiya Biomedical (Seattle, WA, USA) as described by Katus et al.^33^.

#### Determination of creatine kinase -MB

Serum creatine kinase-MB activity was measured using the Spinreact colorimetric kit (Sant Esteve de Bas, Girona, Spain) according to the manufacturer’s protocol. Serum and heart creatine kinase-MB activity was measured using the Spinreact colorimetric kit (Sant Esteve de Bas, Girona, Spain) according to the manufacturer’s protocol.

### Determination of myoglobin

Serum myoglobin measurements were performed using technology provided by BioVision (Milpitas, CA, USA).

### Serum and heart electrolytes

Cardiac tissue electrolyte as sodium and calcium and Serum sodium, potassium, chloride, zinc, and calcium levels were measured using Spectrum kits in Cairo, Egypt, according to the methods described by Trinder^34^, Sunderman^35^, Skeggs and Hochstrasser^36^, Hayakawa^37^, and Gindler^38^, respectively.

### Gene expression of NF-κB (qRT-PCR)

#### Quantitative RT-PCR analysis

The expression of gene of NF-κB was assessed in heart homogenate using quantitative RT-PCR technique. Extraction of total RNA from heart tissue was done by using Qiagen tissue extraction kit (Qiagen Inc., Valencia, USA) according to the manufacturers’ instructions. Determination of the purity and concentration of RNA were obtained spectrophotometrically at 260/280 nm. Then, the extracted RNA was reverse transcribed into complementary DNA using Superscript Reverse Transcription system (Life Technologies Inc., Grand Island, USA) according to the manufacturers’ guidelines. The expression level of NF-κB was analyzed using SYBR Green PCR Master Mix (Applied Bio systems, Foster City, USA) as figured by the manufacturer. The relative expression of target genes was calculated using the 2^−ΔΔCT^ formula using β-actin as the housekeeping gene^39^. The sequences of primers used were as follows:

NF-κB (accession number: NM_199267.2),

- F: 5′- CTGGCCATGGACGATCTGTT − 3,
- R: 5′- GCACTTGTAACGGAAACGCA − 3,

and β-actin (accession number: NM_031144.3,

- F: 5′- TCTGTGTGGATTGGTGGCTC − 3,
- R: 5′- ACGCAGCTCAGTAACAGTCC − 3.

### Normalization of cardiac tissue assays

Approximately 0.1 g of heart tissue was homogenized in 1 mL of ice-cold buffer. The obtained homogenates were used for the determination of antioxidant enzymes, oxidative stress markers, inflammatory markers, Na⁺/K⁺-ATPase, MCP-1, CK-MB, Na⁺, and Ca²⁺. All biochemical results from cardiac homogenates were normalized to tissue weight and expressed per g tissue.

### Histological and morphometric examination

Cardiac tissue was fixed, dehydrated in graded ethanol concentrations, cleared in xylene, embedded in paraffin, sectioned, and stained with hematoxylin and eosin (H&E) as characterized by Svahn and Bancroft^40^. Sections were then examined under a light microscope.

The myocyte cross-sectional area was determined for at least 100 myocytes per hematoxylin and eosin-stained slide. The myocyte cross-sectional area measurements were obtained from digitized image (40x magnification lens). myocyte cross sectional area was measured using a digitizing pad, and the selected cells were cut transversely with the nucleus clearly identified in the center of the myocyte.

### Statistical test

The collected data were statistically tested using GraphPad Prism software (version 5.04, GraphPad Software Inc., La Jolla, CA, USA) by one-way analysis of variance followed by Tukey’s post hoc test. Results are expressed as mean ± standard error, and significant data were recorded at *P* ≤ 0.05.

## Results

### Body weight

**Fig. 1:**
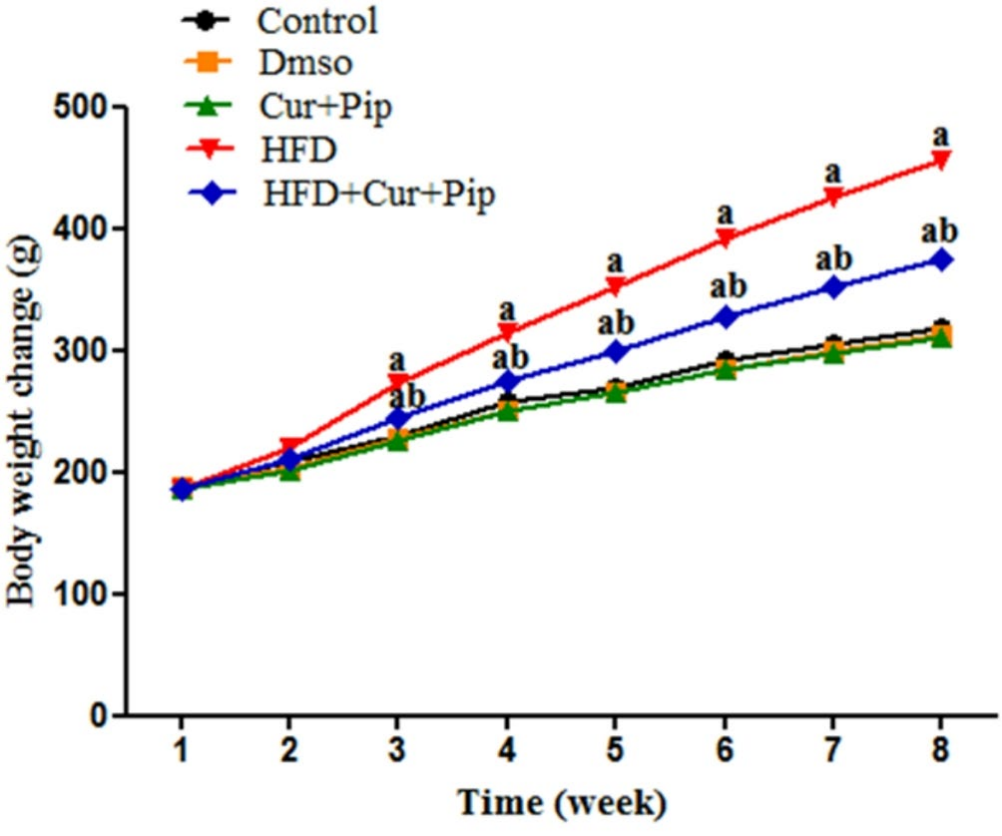
Body weight variations (g) in control and various treatment groups. (**a**) Statistically significant difference relative to the control group (*P* ≤ 0.05). (**b**) Statistically significant difference relative to the HFD group (*P* ≤ 0.05).

The data obtained showed that the body weight of HFD rats increased significantly (*P* ≤ 0.05) (by 40.3%) compared with the control group (21.4%) from the third week of HFD administration. The body weight of the HFD + CUR+PIP treated group was significantly decreased (*P* ≤ 0.05) compared with the HFD group, also from the third week of HFD administration. However, no significant change was observed when CUR + PIP was administered to normal rats (Fig. 1).

### Lipid profile

Animals fed HFD showed a significant increase (*P* ≤ 0.05) in serum TC (115.4%), TG (95.7%), and LDL (348.6%), accompanied by a significant decrease (*P* ≤ 0.05) in HDL levels (51.8%) compared to the control group. However, the HFD + CUR+PIP group showed a significant decrease (*P* ≤ 0.05) in serum TC (31.60%), TG (32.13%), and LDL (43.99%), accompanied by a significant increase (*P* ≤ 0.05) in HDL levels (54.76%) compared to the HFD group. At the same time, no significant increase was observed between the CUR + PIP group and the DMSO group compared to the control group. The results showed that the cardiovascular risk indexes, CRI-I, CRI-II, and AC values significantly increased (*P* ≤ 0.05) in animals fed an HFD compared with the control group (353.1%, 834.2%, and 677.9%, respectively). Meanwhile, the CRI-I, CRI-II, and AC values significantly decreased (*P* ≤ 0.05) in the HFD + Cur+Pip group compared with the HFD rats, showing a significant improvement (56.7%, 64.6%, and 63.4%, respectively). However, no significant changes were observed between the CUR + PIP group, the DMSO group, and the control group (Table 1).

**Table 1:** Serum lipid profiles across control and treated rat groups.

Parameters	Groups				
	Control	DMSO	Cur + Pip	HFD	HFD + Cur+Pip
Cholesterol (mg/dl)					
Mean ± SE	181.2 ± 6.644	183.2 ± 5.553	182.8 ± 3.99	390.4 ± 26.49 ^a^	267.0 ± 18.60^ab^
% of change					
1		+ 1.103	+ 0.883	+ 115.452	+ 47.351
2					− 31.608
Triglycerides (mg/dl)					
Mean ± SE	145.0 ± 6.205	144.6 ± 4.343	148.0 ± 5.967	283.8 ± 6.733 ^a^	192.6 ± 5.963 ^ab^
% of change					
1		− 0.275	+ 2.069	+ 95.724	+ 32.827
2					− 32.135
HDL (mg/dl)					
Mean ± SE	87.20 ± 4.017	86.20 ± 1.655	86.40 ± 1.435	42.00 ± 3.536^a^	65.00 ± 2.302 ^ab^
% of change					
1		− 1.146	− 0.917	− 51.834	− 25.458
2					+ 54.761
LDL (mg/dl)					
Mean ± SE	65.00 ± 9.377	68.08 ± 6.251	66.80 ± 4.552	291.6 ± 25.20 ^a^	163.3 ± 17.81 ^ab^
% of change					
1		+ 4.738	+ 2.769	+ 348.615	+ 151.230
2					− 43.9986
CRI-I (TC/HDL)					
Mean ± SE	2.09 ± 0.13	2.12 ± 0.09	2.11 ± 0.04	9.47 ± 0.78 ^a^	4.10 ± 0.26 ^ab^
% of change					
1		+ 1.43	+ 0.95	+ 353.1	+ 96.1
2					− 56.7
CRI-II (LDL/HDL)					
Mean ± SE	0.76 ± 0.13	0.79 ± 0.08	0.76 ± 0.05	7.10 ± 0.71 ^a^	2.51 ± 0.26 ^ab^
% of change					
1		+ 3.94	+ 0.0	+ 834.2	+ 230.2
2					− 64.6
AC (TC-HDL)/HDL					
Mean ± SE	1.09 ± 0.13	1.12 ± 0.09	1.11 ± 0.04	8.48 ± 0.78^a^	3.10 ± 0.26^ab^
% of change					
1		+ 2.75	+ 1.83	+ 677.9	+ 184.4
2					− 63.4

Data are presented as mean ± SE (*N* = 6). a: Significant differences when compared to the control group (*P* ≤ 0.05). b: Significant differences when comparing HFD + Cur+Pip to the HFD group (*P* ≤ 0.05). (^1^%): Percentage change compared to the control group. (^2%^): Percentage change compared to the HFD group.

### Insulin level

**Fig. 2:**
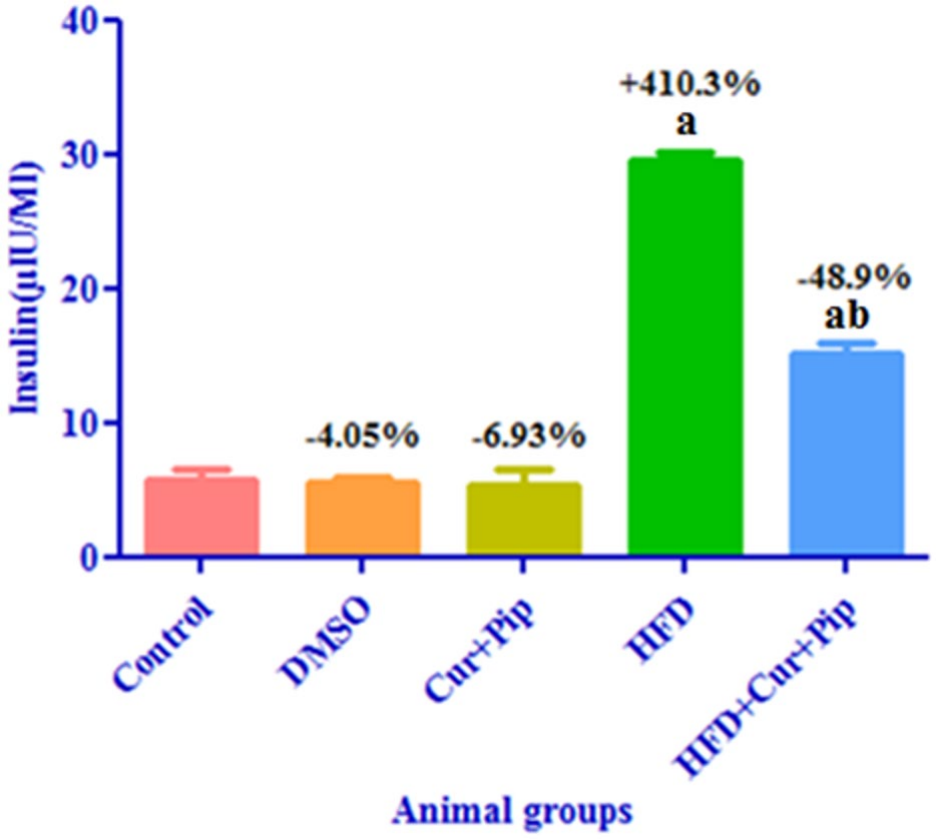
Serum insulin levels in control and various treatment groups. a: Significant difference compared to the control group (*P* ≤ 0.05). b: Significant difference compared to the HFD group (*P* ≤ 0.05).

As shown in Fig. 2, the levels of serum insulin in the HFD group were significantly increased (*P* ≤ 0.05) compared to the control group (by 410.3% increase). Meanwhile, the HFD + CUR+PIP group showed a significant decrease (*P* ≤ 0.05) in serum insulin levels (48.9%) compared to the HFD group. Additionally, no significant difference was observed between the CUR + PIP group and the DMSO group compared to the control group.

### Antioxidant and oxidative stress

HFD group showed a significant decrease (*P* ≤ 0.05) in cardiac antioxidants; GPx (by 37.2%), SOD (by 41.9%), and CAT (by 52.1%) compared to the control rats, accompanied by a significant increase (*P* ≤ 0.05) in oxidative stress markers; MDA (by 124.3%), NO (by 241.0%), and ROS (by 217.3%). However, the HFD + CUR + PIP group showed significant increase (*P* ≤ 0.05) in cardiac antioxidants GPx (by 38.8%), SOD (by 42.2%), and CAT (by 51.7%), and significant decreases (*P* ≤ 0.05) in oxidative stress markers MDA (by 35.1%), NO (by 52.0%), and ROS (by 55.5%) compared to the HFD group. Meanwhile, no significant changes were detected in the above antioxidants (GPx, SOD, and CAT) in the CUR + PIP and DMSO groups compared to the control group. Additionally, no significant changes were observed in oxidative stress markers (MDA, NO, and ROS) in the CUR + PIP and DMSO groups compared to the control rats’ group (Table 2).

**Table 2:** Antioxidants and oxidative stress markers in heart of control and different treated rat groups.

Parameters	Groups				
	Control	DMSO	Cur + Pip	HFD	HFD + Cur+Pip
GPx (U/g)					
Mean ± SE	682.4 ± 6.933	680.2 ± 4.903	684.2 ± 4.620	428.4 ± 4.915^**a**^	595.0 ± 6.000^**ab**^
% of change					
1		− 0.322	+ 0.263	− 37.221	− 12.807
2					+ 38.888
SOD (U/g)					
Mean ± SE	185.0 ± 5.950	187.6 ± 7.461	182.0 ± 4.301	107.4 ± 6.911^**a**^	152.8 ± 4.079^**ab**^
% of change					
1		+ 1.405	− 1.621	− 41.945	− 17.405
2					+ 42.271
CAT (U/g)					
Mean ± SE	193.2 ± 9.107	192.4 ± 6.250	190.0 ± 5.301	92.40 ± 6.329^**a**^	140.2 ± 5.453^**ab**^
% of change					
1		− 0.414	− 1.656	− 52.173	− 27.432
2					+ 51.731
MDA (nmol/g)					
Mean ± SE	252.8 ± 12.54	250.4 ± 11.00	250.4 ± 6.218	567.2 ± 13.77^**a**^	367.8 ± 12.16^**ab**^
% of change					
1		− 0.949	− 0.949	+ 124.367	+ 45.490
2					− 35.155
NO (mmol/g)					
Mean ± SE	25.80 ± 2.782	26.20 ± 1.744	25.20 ± 2.267	88.00 ± 3.564^**a**^	42.20 ± 4.067^**ab**^
% of change					
1		+ 1.550	− 2.325	+ 241.085	+ 63.565
2					− 52.045
ROS (nmol/g)					
Mean ± SE	1.537 ± 0.06173	1.543 ± 0.02963	1.580 ± 0.08185	4.877 ± 0.069^**a**^	2.170 ± 0.097^**ab**^
% of change					
1		+ 0.390	+ 2.797	+ 217.306	+ 41.184
2					− 55.505

Data are presented as mean ± SE (*N* = 6). a: Significant differences when compared to the control group (*P* ≤ 0.05). b: Significant differences when comparing HFD + Cur+Pip to the HFD group (*P* ≤ 0.05). (^1^%): Percentage change compared to the control group. (^2^%): Percentage change compared to the HFD group.

### Na+/K+-ATPase activity

**Fig. 3:**
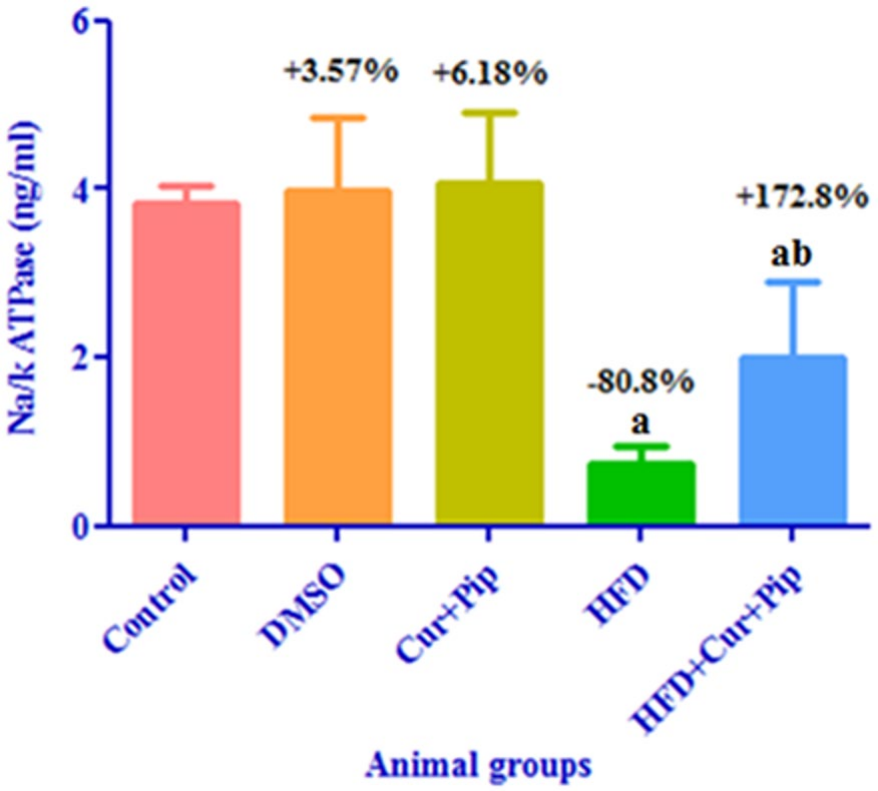
Na+/K+-ATPase activity in cardiac tissue of control and various treatment groups. (**a**) Significant difference compared to the control group (*P* ≤ 0.05). (**b**) Significant difference compared to the HFD group (*P* ≤ 0.05).

As shown in Fig. 3, the HFD group showed a significant decrease (*P* ≤ 0.05) in myocardial Na+/K+-ATPase activity (by 80.8%) compared to the control group. However, the HFD + CUR+PIP group showed a significantly increased (*P* ≤ 0.05) (by 172.8%) compared to the HFD group. In addition, no significant changes were observed in the CUR + PIP and DMSO groups compared to the control group.

### Inflammatory markers

**Fig. 4:**
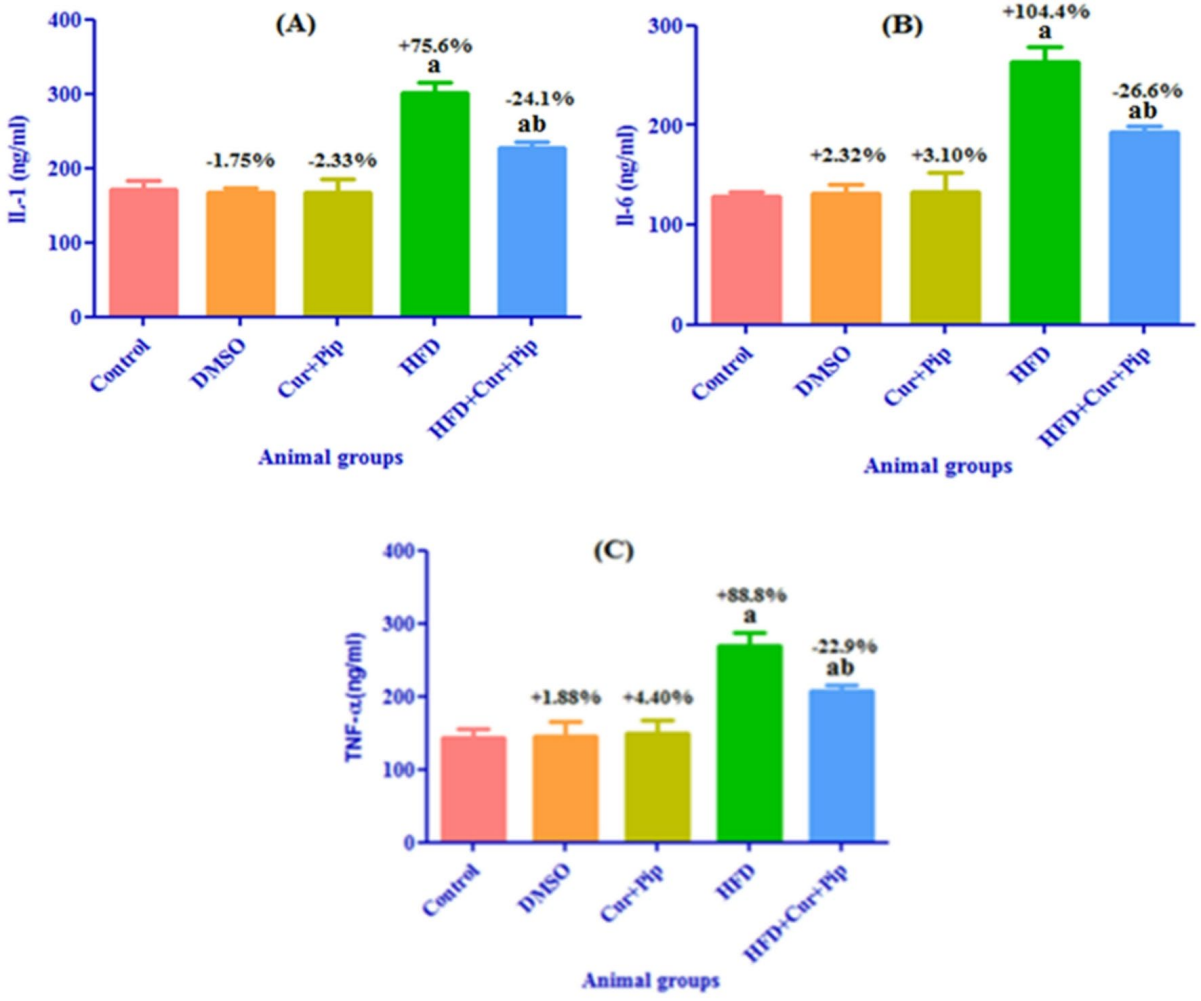
Inflammatory markers in the heart: IL-1 (**A**), IL-6 (**B**), and TNF-α (**C**) in control and various treatment groups. a: Significant difference compared to the control group (*P* ≤ 0.05). b: Significant difference compared to the HFD group (*P* ≤ 0.05).

As shown in Fig. 4 “A, B, C”, HFD rats showed a significant increase (*P* ≤ 0.05) in cardiac inflammatory markers; IL-1 (by 75.6%) (Fig. 4 “A”), IL-6 (by 104.4%) (Fig. 4 “B”), and TNF-α (by 88.8%) (Fig. 4"C”) compared to the control group. Meanwhile, the HFD + Cur+Pip group showed a significant decrease (*P* ≤ 0.05) in cardiac inflammatory markers; IL-1 (by 24.1%) (Fig. 4 “A”), IL-6 (by 26.6%) (Fig. 4 “B”), and TNF-α (by 22.9%) (Fig. 4 “C”) compared to the HFD group. Meanwhile, no significant changes were detected in the above cardiac inflammatory markers between the Cur + Pip and DMSO groups compared with the control group.

### C-reactive protein (CRP), monocyte chemoattractant protein-1 (MCP-1), plasma plasminogen activator inhibitor-1 (PAI-1), and adiponectin levels

As shown in Table 3, HFD rats showed a significant increase (*P* ≤ 0.05) in serum CRP (by 238.0%), MCP-1 (by 46.8%), and PAI-1 levels (by 257.2%), accompanied by a significant decrease (*P* ≤ 0.05) in adiponectin levels (by 70.6%) when compared to the control rats. However, the HFD + CUR + PIP group showed a significant decrease (*P* ≤ 0.05) in serum CRP (by 52.3%), MCP-1 (by 19.3%), and PAI-1 (by 36.9%), accompanied by a significant increase (*P* ≤ 0.05) in adiponectin levels (by 134.4%) compared to the HFD rats. No significant differences were observed in the levels of CRP, MCP-1, PAI-1, and adiponectin between the CUR + PIP and DMSO groups and the control group.

**Table 3:** C-reactive protein (CRP), monocyte chemoattractant protein-1 (MCP-1), plasma plasminogen activator inhibitor-1(PAI-1), and adiponectin levels in control and treated rat groups, reported as mean ± SE (*N* = 6).

Parameters	Groups				
	Control	DMSO	Cur + Pip	HFD	HFD + Cur+Pip
CRP (mg/L)					
Mean ± SE	14.30 ± 1.20	13.64 ± 0.70	13.42 ± 0.55	48.34 ± 3.23 ^**a**^	23.04 ± 1.71 ^**ab**^
% of change					
1		− 4.615	− 6.153	+ 238.0	+ 61.11
2					− 52.33
MCP-1 (ng/g tissue)					
Mean ± SE	190.0 ± 7.37	191.0 ± 11.59	190.7 ± 12.57	279.0 ± 10.02 ^**a**^	225.0 ± 10.44 ^**ab**^
% of change					
1		+ 0.526	+ 3.684	+ 46.842	+ 18.421
2					− 19.354
PAI-1 (Pg/ml)					
Mean ± SE	0.843 ± 0.08	0.806 ± 0.07	0.833 ± 0.07	3.013 ± 0.04 ^**a**^	1.90 ± 0.07 ^**ab**^
% of change					
1		− 4.340	− 1.185	+ 257.28	+ 125.30
2					− 36.93
Adiponectin (µg/ml)					
Mean ± SE	29.0 ± 1.85	29.7 ± 2.57	29.9 ± 3.59	8.50 ± 0.87 ^**a**^	19.93 ± 1.43 ^**ab**^
% of change					
1		+ 2.413	+ 3.103	− 70.689	-31.27
2					+ 134.47

a: Significant differences when compared to the control group (*P* ≤ 0.05). b: Significant differences when comparing HFD + CUR+PIP to the HFD group (*P* ≤ 0.05). (^1^%): Percentage change compared to the control group. (^2^%): Percentage change compared to the HFD group.

### Cardiac biomarkers

**Fig. 5:**
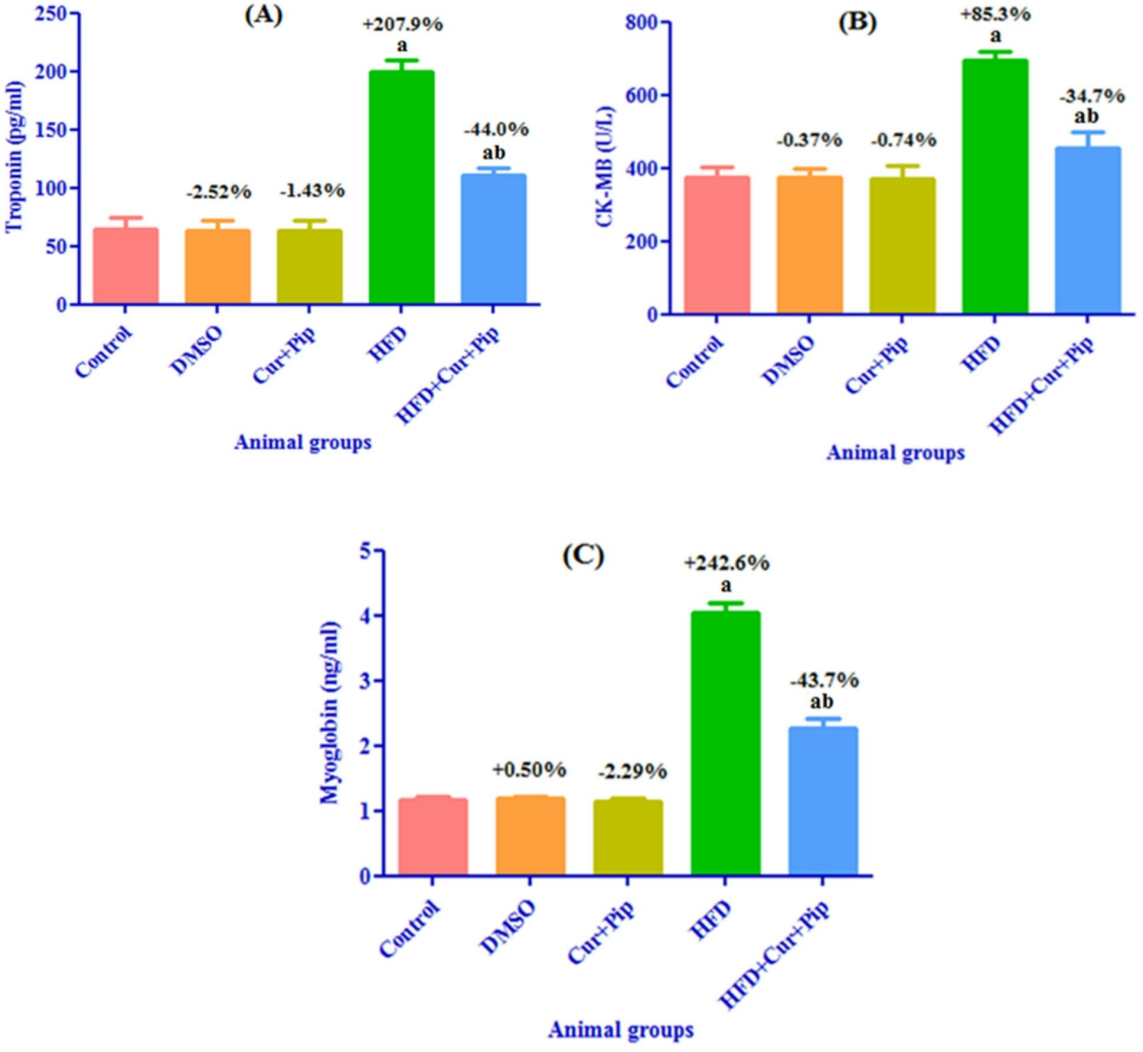
Cardiac biomarkers in serum: troponin (**A**), CK-MB (**B**), and myoglobin (**C**) in control and various treatment groups. a: Significant difference compared to the control group (*P* ≤ 0.05). b: Significant difference compared to the HFD group (*P* ≤ 0.05).

Animals fed on HFD showed a significant increase (*P* ≤ 0.05) in cardiac biomarkers troponin T (by 207.9%) (Fig. 5 “A”), CK-MB (by 85.3%) (Fig. 5 “B”), and myoglobin (by 242.6%) (Fig. 5 “C”) compared to the control group. Meanwhile, the HFD + CUR+PIP group showed a significant decrease (*P* ≤ 0.05) in cardiac biomarkers troponin T (by 44.0%) (Fig. 5 “A”), CK-MB (by 34.7%) (Fig. 5 “B”), and myoglobin (by 43.7%) (Fig. 5 “C”) when compared to the HFD rats. Likewise, no significant changes were observed between the CUR + PIP and DMSO groups when compared to the control group for the above-mentioned cardiac biomarkers (Figs. 5 “A, B, C”).

### Electrolyte balance in serum and cardiac tissue

#### Serum electrolytes

HFD group showed a significant decrease (*P* ≤ 0.05) in serum K+ (by 69.1%), Ca (by 40.0%), and Zinc (by 68.1%) concentrations, accompanied with a significant increase (*P* ≤ 0.05) in serum Na+ (by 45.1%) and Cl- (by 54.3%) concentrations when compared to the control group. However, HFD + CUR + PIP group showed a significant increase (*P* ≤ 0.05) in serum K+ (by 113.4%), Ca (by 35.7%), and Zinc (by 137.3%) concentrations accompanied with a significant decrease (*P* ≤ 0.05) in serum Na+ (by 14.6%) and Cl- (by 21.1%) concentrations when compared to the HFD rats. On the other hand, no significant variation was observed in the above serum electrolytes (Na+, K+, Cl-, Ca, and Zinc) between the CUR + PIP group and the DMSO group when compared to the control rats (Table 4).

**Table 4:** Serum electrolyte concentrations in control and treated rat groups, reported as mean ± SE (*N* = 6).

Parameters	Groups				
	Control	DMSO	Cur + Pip	HFD	HFD + Cur+Pip
Na^+^ (mmol/L)					
Mean ± SE	130.6 ± 4.445	128.8 ± 3.707	128.2 ± 2.764	189.6 ± 4.445 ^**a**^	161.8 ± 3.707 ^**ab**^
% of change					
1		− 1.378	− 1.837	+ 45.176	+ 23.889
2					− 14.662
K^+^ (mg/dL)					
Mean ± SE	5.90 ± 0.450	6.18 ± 0.3169	6.02 ± 0.602	1.81 ± 0.243 ^**a**^	3.880 ± 0.528 ^**ab**^
% of change					
1		+ 4.745	+ 2.033	− 69.186	− 34.237
2					+ 113.421
Cl^−^ (mg/dL)					
Mean ± SE	82.80 ± 6.264	79.60 ± 2.159	83.80 ± 2.223	127.8 ± 4.140 ^**a**^	100.8 ± 6.264 ^**ab**^
% of change					
1		− 3.864	+ 1.207	+ 54.347	+ 21.739
2					− 21.126
Ca (mg/dL)					
Mean ± SE	10.64 ± 0.461	10.46 ± 0.252	10.76 ± 0.258	6.380 ± 0.208 ^**a**^	8.660 ± 0.280 ^**ab**^
% of change					
1		− 1.691	+ 1.127	− 40.037	− 18.609
2					+ 35.736
Zinc (µg/dL)					
Mean ± SE	129.4 ± 3.458	128.2 ± 3.338	130.8 ± 4.737	41.20 ± 2.746 ^**a**^	97.80 ± 4.737 ^**ab**^
% of change					
1		− 0.927	+ 1.081	− 68.160	− 24.420
2					+ 137.378

a: Significant differences when compared to the control group (*P* ≤ 0.05). b: Significant differences when comparing HFD + CUR+PIP to the HFD group (*P* ≤ 0.05). (^1^%): Percentage change compared to the control group. (^2^%): Percentage change compared to the HFD group.

### Cardiac tissue electrolyte

#### Sodium (Na⁺) level in cardiac tissue

**Fig. 6:**
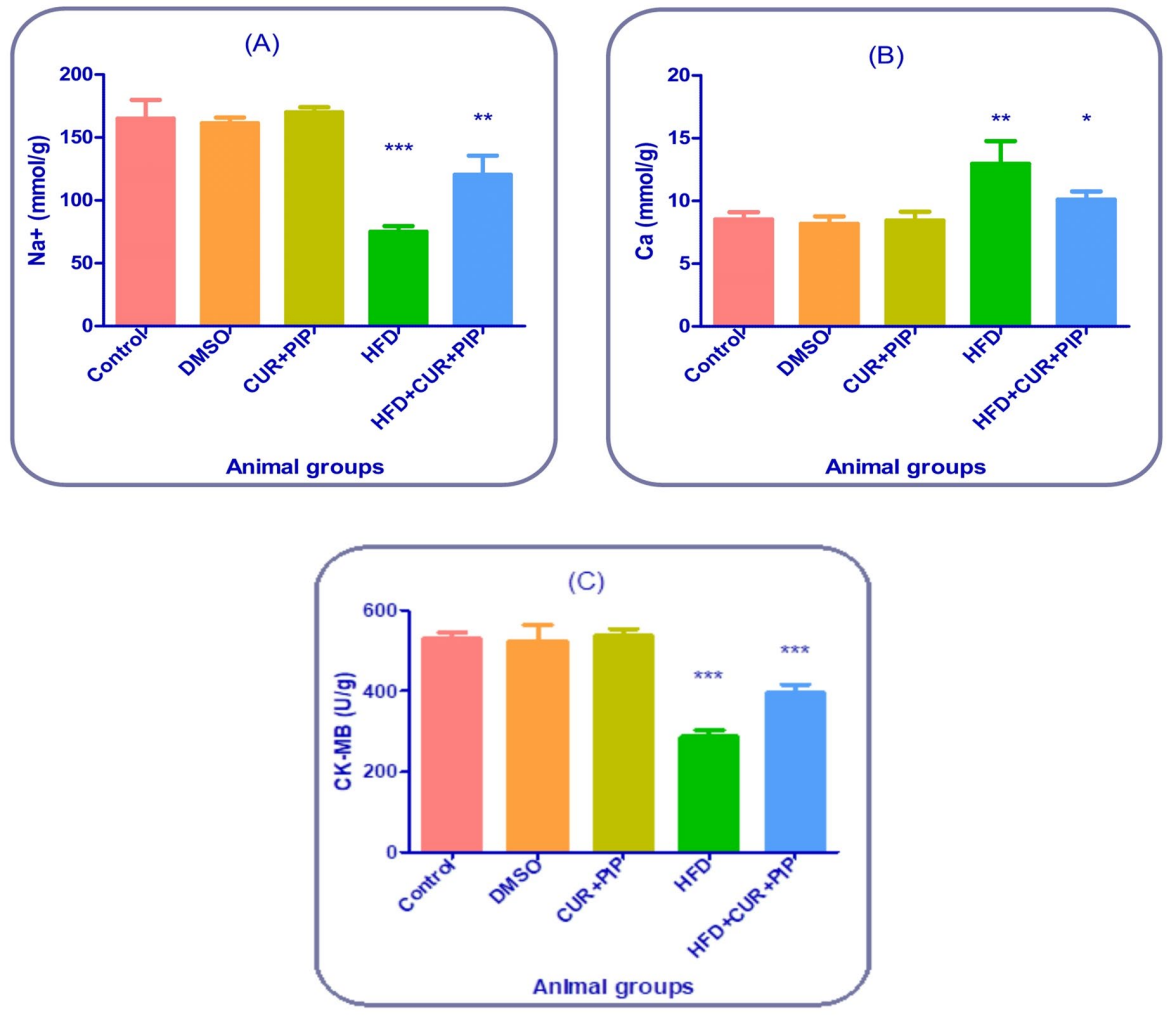
Cardiac biomarkers and electrolyte in heart tissue: Na ^+^ (**A**), Ca (**B**), and CK-MB (**C**) in control and various treatment groups. a: Significant difference compared to the control group (*P* ≤ 0.05). b: Significant difference compared to the HFD group (*P* ≤ 0.05).

Cardiac Na⁺ concentration showed a significant decrease of about **37%** in the HFD group compared to the control rats, indicating ionic imbalance due to obesity-induced cardiac injury. Treatment with curcumin + piperine markedly improved Na⁺ levels, showing an increase of approximately **60%** compared to the HFD group, suggesting partial normalization of sodium homeostasis and restoration of cardiac ionic balance (Fig. 6 “A”).

#### Calcium (Ca²⁺) level in cardiac tissue

Cardiac Ca²⁺ levels were significantly elevated by about **55–60%** in HFD-fed rats compared with the control group, reflecting calcium overload and disturbed cardiomyocyte function. Administration of curcumin + piperine reduced tissue calcium concentration by approximately **30%** compared to the HFD group, indicating attenuation of Ca²⁺ accumulation and improved cardiomyocyte stability (Fig. 6 “B”).

#### Cardiac tissue CK-MB activity

In cardiac tissue homogenates, CK-MB activity significantly decreased by approximately **46%** in the HFD group compared to the control, confirming myocardial cell damage and enzyme leakage. Treatment with curcumin + piperine markedly restored CK-MB activity, showing an improvement of about **38%** compared to the HFD group. These results indicate that curcumin combined with piperine preserved cardiac membrane integrity and reduced myocardial injury induced by high-fat diet (Fig. 6 “C”).

### NF-κB gene expression in cardiac tissue

**Fig. 7:**
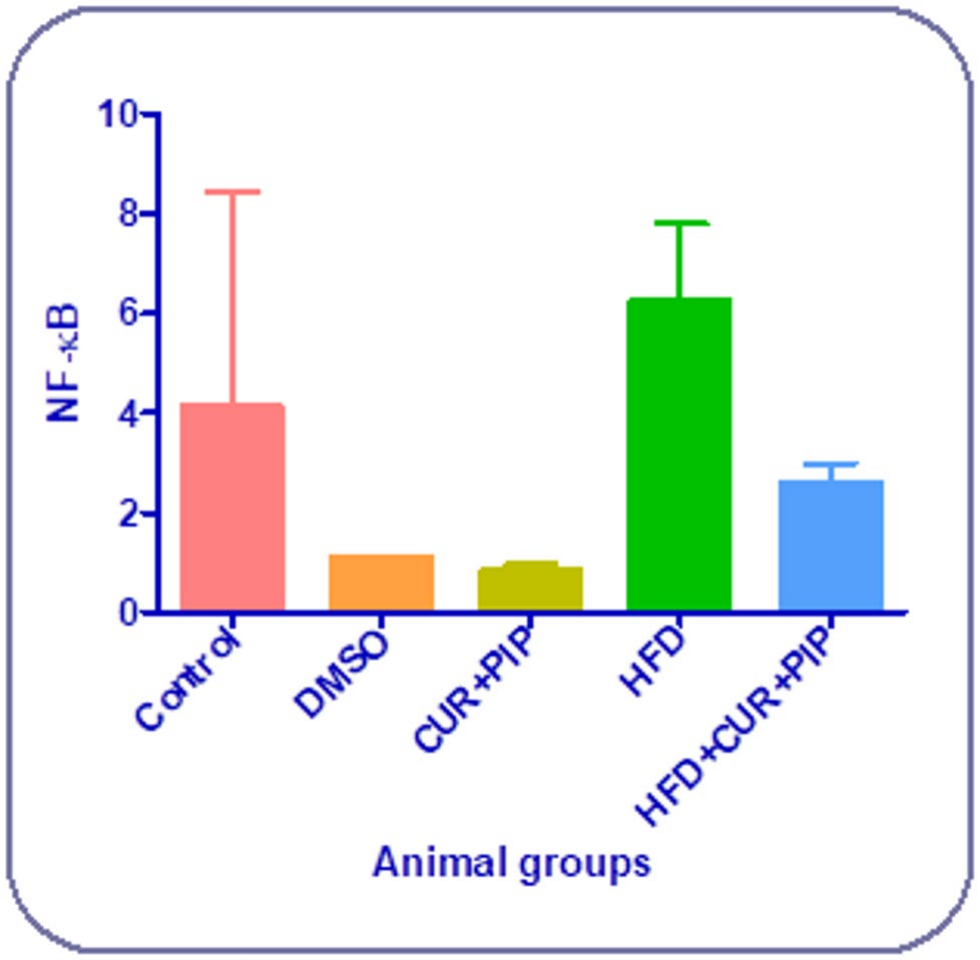
Results NF-κB relative expression in control and various treatment groups. a: Significant difference compared to the control group (*P* ≤ 0.05). b: Significant difference compared to the HFD group (*P* ≤ 0.05).

The relative mRNA expression of NF-κB in cardiac tissue showed a significant up-regulation in HFD-fed rats compared to the control group, with an increase of about 530%. Treatment with curcumin + piperine markedly downregulated NF-κB expression by approximately 59% compared to the HFD group. Both the control, DMSO, and CUR + PIP groups exhibited nearly baseline expression levels, indicating that the combined treatment effectively suppressed HFD-induced NF-κB activation in cardiac tissue (Fig. 7).

### Histological examination

**Plate 1.**
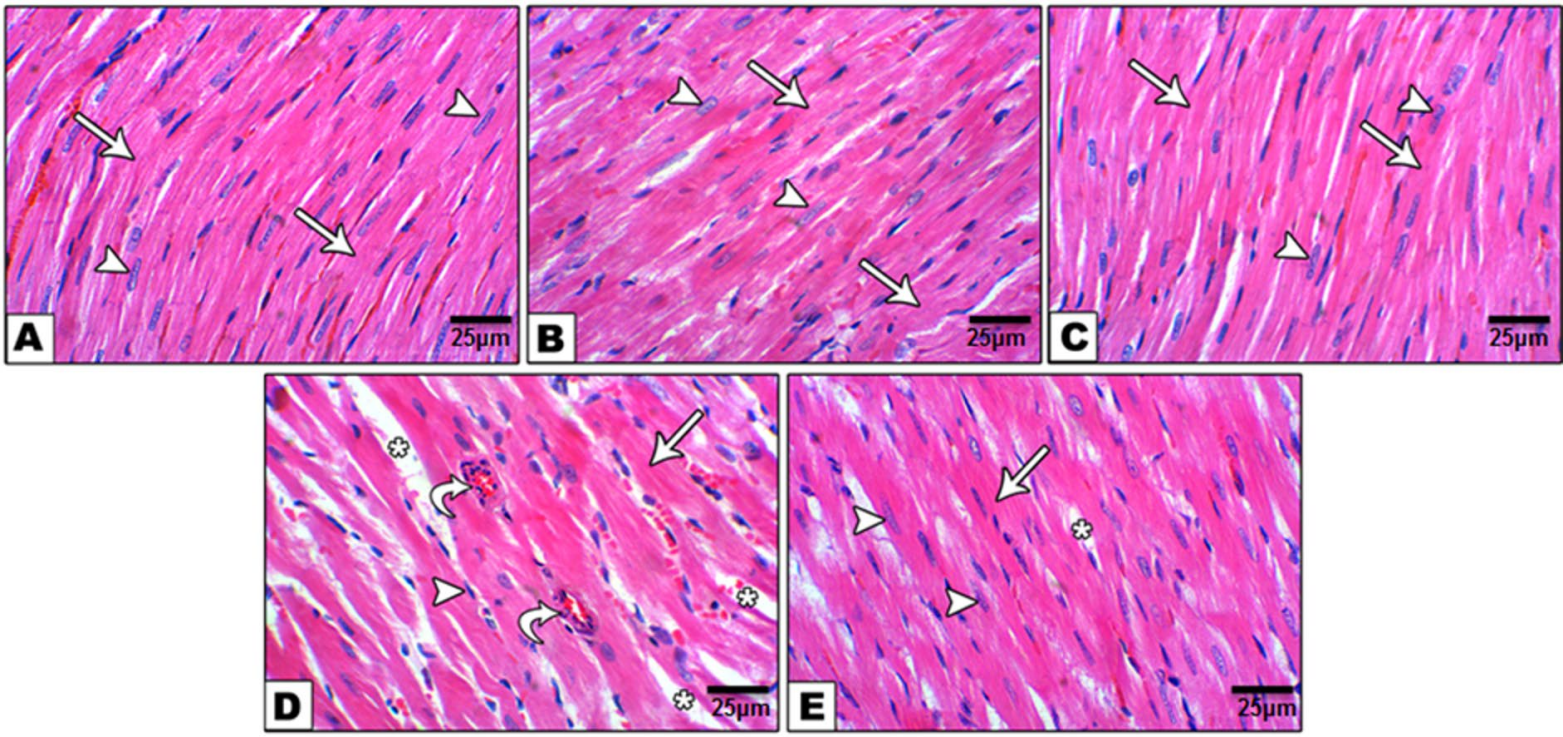
Photomicrograph of hematoxylin and eosin (H&E) stained heart sections in control (**A**), DMSO (**B**), and curcumin plus piperine (**C**) groups showed normal appearance and well organization of cardiac muscle fibers (arrow) with minimal interstitial space and intact intercalated discs containing connective tissue and blood vessels, acidophilic sarcoplasm and centrally located nuclei (arrowhead). Cardiac section from HFD group (**D**) showing markedly increased interstitial space (asterisk) with degenerated muscle fibers (arrow), inflammatory cell infiltration and congested blood vessels (curved arrow), and pyknotic nuclei (arrowhead). However, HFD rats treated with curcumin plus piperine (**E**) showing markedly decreased interstitial space (asterisk) with increased size of muscle fibers (arrow) and presence of centrally located nuclei (arrowhead).

Heart sections stained by H&E in the control, DMSO & curcumin + piperine groups showed normal appearance and well organization of cardiac muscle fibers (arrow) with minimal interstitial space & intact intercalated discs comprising connective tissue & blood vessels, acidophilic sarcoplasm & centrally located nuclei (arrowhead) (Plate 1 “A, B, C”). HFD group showed markedly increase interstitial space (asterisk) with degenerated muscle fibers (arrow), inflammatory cell infiltration & congested blood vessels (curved arrow), and pyknotic nuclei (arrowhead) (Plate 1 “D”). HFD group showed markedly increase in interstitial space (asterisk) with degenerated muscle fibers (arrow), inflammatory cell infiltration & congested blood vessels (curved arrow), and pyknotic nuclei (arrowhead) (Plate 1 “D”). Otherwise, administration of curcumin + piperine with a high-fat diet showed a remarkable decrease in the interstitial space (asterisk) with increased size of muscle fibers (arrow) and presence of centrally located nuclei (arrowhead) (Plate 1"E”). Myocyte cross sectional area in the myocardia of the HFD group was 455.5 ± 13.7µm^2^, which were significantly more than those of control, DMSO, CUR + PIP, HFD + CUR+PIP groups (245.5 ± 10.3µm2, 250.0 ± 13.6µm2, 293.6 ± 12.5µm2 respectively).

### Morphometric analysis

**Fig. 8:**
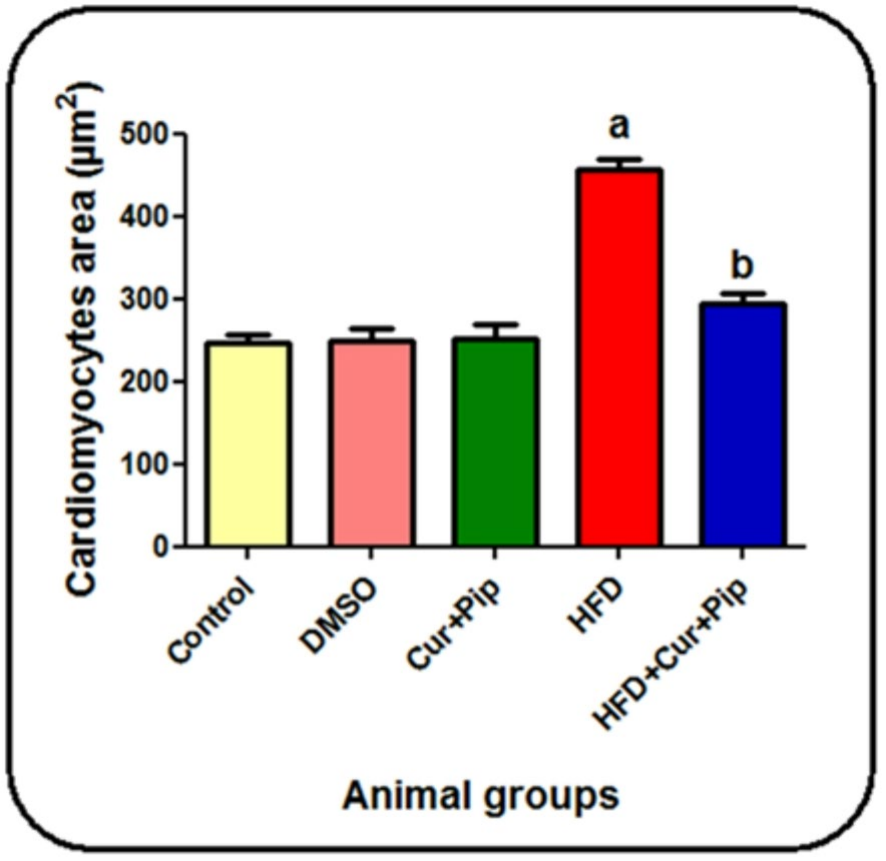
morphometrical analysis of cardiomyocytes cross sectional area (µm2) in control and different treatment groups. (a): significant difference compared to control group (*p* < 0.05). (b): significant difference compared to the HFD.

Morphometric evaluation of the cardiomyocytes’ cross-sectional area was carried out to quantitatively assess the histological changes among the experimental groups. As shown in Figure 8, the HFD group exhibited a significant increase in myocyte area compared to the control, DMSO, and CUR + PIP groups, indicating hypertrophy and structural disorganization of cardiac fibers due to obesity-induced stress. Treatment with curcumin and piperine markedly reduced myocyte size compared to the HFD group, suggesting improved myocardial architecture and restoration of normal cellular dimensions.

**Fig. 9:**
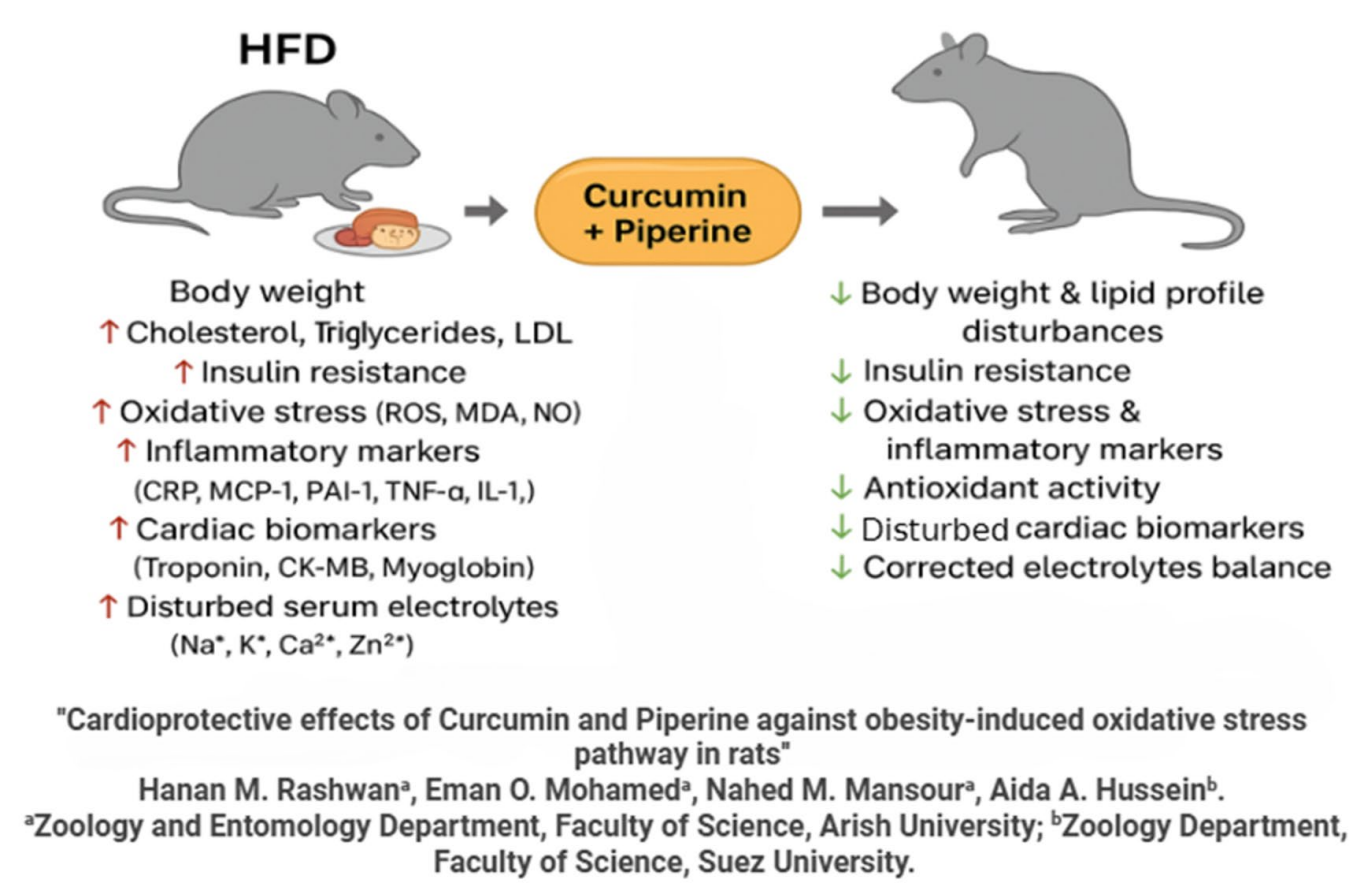
Graphical summary illustrating the overall experimental design and the proposed protective effects of curcumin + piperine against HFD-induced cardiac dysfunction.

Finally, a graphical summary illustrating the experimental design and the proposed molecular mechanism underlying the cardioprotective effects of curcumin and piperine against HFD-induced cardiac dysfunction is presented in Fig. 9.

## Discussion

An imbalance between energy intake and expenditure can lead to excessive fat accumulation in the body, particularly in metabolic diseases such as obesity, which can have adverse health effects^41^. Even when considering all modifiable factors like diet and lifestyle, obesity is correlated with various causes, including genetic predisposition and environmental factors^42^. Overweight and Obesity are widespread problems in developing countries and are associated with increased risks of adverse health outcomes^43^. The WHO estimates that over 1.6 billion individuals (aged 15 years and older) are described as overweight. It is predicted that 51% of the world’s people will be obese in 2030^8^. This research aims to discuss the association between oxidative stress, inflammation, immunological changes, metabolic abnormalities, and other risk factors identified by histopathological findings and obesity-induced cardiotoxicity. This study also examines the impact of the CUR + PIP combination treatment on reducing the risk of obesity and being overweight. This study also examines the impact of the CUR + PIP combination treatment on reducing the risk of obesity and being overweight. In this research, rats were administered an HFD for 8 weeks to produce obesity. This treatment resulted in body weight gain in HFD rats compared to the control rats. However, the HFD + CUR + PIP group showed a significant decrease in body weight when compared to the HFD group. Many studies have linked long-term HFD intake to body weight gain, and these effects may be due to a slower accumulation of body fat and an increase in fat mass^44^.

Rats fed HFD for 2 months developed obesity with dyslipidemia, evidenced by increased TC, TG, LDL, and decreased HDL levels, consistent with previous studies^45,46^. These changes may reflect impaired lipid metabolism associated with HFD intake. Approximately 60–80% of cholesterol is transported by LDL-C, the primary carrier of cholesterol in the blood. Some of the cholesterol is used by tissues, while the rest is used by the liver. However, high LDL-C levels in the blood can lead to cholesterol accumulation^47^. However, HDL-C can absorb cholesterol through a process called reverse cholesterol transport, transporting it back to the liver for disposal or processing. Therefore, lowering HDL-C levels is associated with an increased risk of CVD and decreased cholesterol removal from extrahepatic tissues^48^. In this research, the combined intake of CUR + PIP by HFD rats showed a significant improvement in lipid levels, indicating a lipid-lowering effect., suggesting the lipid-lowering effect of Cur and Pip. The results of this study align with those of previous research^49,50^. In previous studies, the combined intake of CUR + PIP significantly decreased serum TG levels, suggesting a triglyceride-lowering effect of CUR. In addition, Seo et al.^51^ revealed that CUR significantly decreased the levels of triglycerides, cholesterol, β-oxidation, fatty acid synthase (FA), and free fatty acids (FFA) in plasma. Comparable results were also reported by Zhou et al.^52^. Previous studies have suggested that the lipid-lowering effect of CUR may involve the modulation of lipid mobilization and absorption, which could contribute to the effects observed in this study. Therefore, Co-administration of CUR + Pip may be the best method to simultaneously increase the bioavailability and circulating lipid-lowering effect of CUR^53^.

Current evidence indicates that chronic consumption of a high-carbohydrate diet increases both basal and postprandial insulin levels, leading to hyperinsulinemia and insulin resistance^54^. A pathophysiological condition known as insulin resistance occurs when the body resists the impacts of insulin on glucose absorption, metabolism, or storage. This resistance leads to compensatory hyperinsulinemia, which results in increased insulin secretion from the pancreas. This can ultimately lead to impaired glucose tolerance^55^. In obesity, insulin resistance is characterized by decreased insulin-stimulated glucose transport and metabolism in skeletal muscle and adipocytes, as well as impaired control of glucose production in liver^56^. Hyperinsulinemia promotes free fatty acid production and inhibits apolipoprotein synthesis in the liver, while insulin resistance leads to impaired insulin regulation of lipolysis. As a result, insulin resistance enhances hepatic lipogenesis and adipose tissue lipolysis, leading to fat accumulation in the liver and hepatic steatosis^57^. Roza et al.^58^ showed that the levels of blood insulin were significantly increased in rats fed on HFD, which may be due to diet-induced insulin resistance. Comparable results were obtained in present research, where serum insulin levels were significantly increased in rats fed on HFD compared to the normal rats. Several studies have suggested that the hypoglycemic effects of CUR alone or in combination with Pip may improve insulin and glucose homeostasis through various mechanisms of action^49^. CUR + PIP showed hypoglycemic effects in HFD rats, which may be related to mechanisms suggested in previous studies, such as enhanced glucose uptake or insulin regulation^49^.

Oxidative stress is a common pathophysiological process in several pathological conditions, including obesity^59^. Excessive fat intake leads to the accumulation of free fatty acids and free sugars in visceral tissues, skeletal muscle, and the liver, contributing to the development of obesity^60^. Oxidation of lipids stored in tissues can release reactive oxygen species (ROS) and other free radicals. ROS promote lipid peroxidation by degrading unsaturated fatty acids in cell membranes and decrease endogenous antioxidants, resulting in oxidative stress-induced tissue damage^58^. In this light, recent studies have revealed a significant decrease in antioxidant enzymes (SOD, CAT, GSH, GPx), TAC, and oxidative stress markers (ROS, MDA, NO) in cardiac tissues of rats fed an HFD diet. Previous studies^58^^59^demonstrated a decrease in tissue antioxidant defenses in the HFD group supporting our findings. Furthermore, Feriani et al.^60^ observed a significant increase in oxidative stress markers, such as MDA, PC, and ROS, and a corresponding decrease in antioxidants, including GSH, SOD, CAT, and GPx, in the cardiac tissues of HFD rats. In this research, we found that oral administration of CUR + PIP to HFD rats significantly increased antioxidant enzymes (SOD, CAT, GSH, GPx) and TAC, while also significantly decreasing oxidative stress indicators (ROS, MDA, NO) in cardiac tissue. The significant increase of antioxidant activity of the combination of CUR + PIP may be attributed to the direct neutralization of reactive oxygen metabolites, such as O2, OH, and nitrogen dioxide radicals, which may be promoted by the ROS-scavenging effects of antioxidant compounds, such as flavonoids, glycosides, polyphenols, steroids, tannins, and triterpenoids, as well as the phenolic hydroxyl groups of CUR^61^. These results support previous studies^62,63^.

The activity of Na+/K+-ATPase decreased in the adipose tissue of obese individuals and was inversely correlated with blood pressure, BMI, and the oral glucose tolerance test^64^. Decreased activity of Na+/K+-ATPase in tissue is associated with obesity and may be related to hyperglycemic hyperinsulinemia, which may inhibit or inactivate the enzyme^65^. In obese rats, a marked increase in oxidative stress, accompanied by a decrease in the activity of cardiac Na+/K+-ATPase, has been observed as a factor accelerating cardiac dysfunction^66^. Na+/K+-ATPase is a protein found in the membrane of higher eukaryotic cells. It actively transports sodium and potassium ions and regulates intracellular calcium concentration by exchanging sodium (Na+) and potassium (K+) ions across the cell membrane^65^In this study, we found that Na + and K+ ions were significantly decreased in HFD rats, and Na+/K+-ATPase function was significantly suppressed in cardiac tissues. Changes in Na+/K+-ATPase activity in HFD rats may affect ion transport and membrane potential, which could contribute to cardiac alterations^67^. In this study, the administration of CUR + PIP was shown to be effective in preventing the high-fat diet-induced decrease in Na+/K+-ATPase activity. The antioxidant effect of CUR + PIP may prevent the inhibition of the activity of Na+/K+- ATPase^68^^69^.

In line with these findings, the alterations detected in both serum and cardiac tissue electrolyte levels in the present study further confirm the impaired ionic regulation associated with obesity. In serum, HFD-fed rats showed a significant decrease in Na⁺ and an increase in Ca²⁺ levels, while in cardiac tissue, Na⁺ content was reduced and Ca²⁺ was markedly accumulated within the myocardium. These findings are consistent with previous reports indicating that obesity and diabetes can downregulate Na⁺/K⁺-ATPase activity and disturb ionic homeostasis in different tissues, including the heart ^64,66^.

Treatment with curcumin combined with piperine significantly restored both serum and tissue electrolyte levels toward normal values, suggesting membrane stabilization and improved ionic transport efficiency within cardiomyocytes. This effect may be attributed to curcumin’s ability to enhance Na⁺/K⁺-ATPase function and maintain Na⁺/Ca²⁺ balance, as previously demonstrated in models of organ toxicity ^5^.

Inflammation is one of the mechanisms underlying the development of CVD in obese humans^70^and obese animals^71^. Obesity is a known risk factor for cardiac damage due to excess fat accumulation in the heart, which results in increased inflammatory responses, as well as infiltration of immune cells into adipocytes^72^. In various states of obesity, adipose tissue-derived proinflammatory cytokines (IL-1, IL-6, TNF-α) and bioactive mediators (CRP, MCP-1, PAI-1, adiponectin), and inflammatory cytokines and bioactive mediators produced by circulating macrophages and monocytes promote cardiac inflammation^73^.

CRP is a protein produced in the liver as a result of inflammatory cytokines like TNF-α and IL-6. It also belongs to the innate immune response protein family. Trauma, infection, and other inflammatory stimuli lead to a rapid increase in CRP concentrations, promoting the clearance of necrotic and apoptotic cells by phagocytes^74^. MCP1 plays a vital role in innate immune and tissue inflammatory processes by stimulating the recruitment and activation of monocytes and macrophages to produce inflammatory factors, such as TNF-α, IL-1, and IL-6^75^. PAI-1 is a strong inhibitor of plasminogen activators and the fibrinolytic system and is known to be increased in several clinical settings with a high prevalence of cardiovascular disease^76^. TNF-α may be linked to the development of CVD, both locally and systemically^77^. TNF-α is one of several protective physiological regulators and a potent agonist of PAI-1 production, playing a crucial role in regulating PAI-1 in various diseases. TNF-α may contribute to increased PAI-1 levels in obese individuals^78^.

Several studies have shown that continuous consumption of HFD for 8–16 weeks can lead to obesity & exacerbation of pro-inflammatory conditions, such as increased levels of the inflammatory marker MCP-1 in Wistar rats^79^. Similarly, our study showed increased cardiac inflammatory markers; IL-1, IL-6, and TNF-α & serum inflammatory markers; CRP, MCP-1, and PAI-1, and decreased serum levels of the anti-inflammatory marker adiponectin in HFD rats, which agree with the findings of Michicotl-Meneses et al.^80^.

Furthermore, recent results show that oral administration of the CUR + PIP combination in HFD group significantly decreases cardiac inflammatory markers, including IL-1, IL-6, and TNF-α, as well as serum inflammatory markers, such as CRP, MCP-1, and PAI-1, while increasing serum levels of the anti-inflammatory marker adiponectin. Thus, combining Cur with Pip decreased inflammatory markers, including IL-1, IL-6, TNF-α, CRP, MCP-1, and PAI-1, suggesting that Cur has a notable anti-inflammatory effect. CUR and PIP showed anti-inflammatory effects in HFD rats. Previous studies suggest that CUR may inhibit the TNF-α and NF-κB pathways^81^and modulate adiponectin levels^82^, which could contribute to the observed anti-inflammatory effects, leading to reduced intracellular inflammation. Another pathway, CUR, has been reported to decrease CRP levels and cause a significant decrease in white blood cell counts^72^. Both of these effects are potential candidates for suppressing enhanced inflammation. Moreover, these results agree with those of previous studies^83^^63^.

CVD is one of the disease risks associated with obesity81. Consumption of HFD causes a significant increase in triglycerides and cholesterol, leading to hyperlipidemia, a significant risk factor for CVD^42^.

As hyperlipidemia progresses, cardiac cells are damaged by increased free fatty acids, which disrupt mitochondrial phosphorylation, thereby generating additional free radicals and exacerbating lipid peroxidation within cardiomyocytes^85^. Alterations in cardiac permeability and cell membrane integrity can lead to the release of various enzymes, including CK-MB, LDH, ALT, and AST, from injured cardiomyocytes into the extracellular fluid.

Our findings showed that the levels of CK-MB, troponin T, and myoglobin were significantly increased in rats fed on a high-fat diet (HFD). These results are findings were consistent with those of Ahmad et al. (2022) 86, who observed that the release of these cardiac biomarkers into the circulation and different markers in many tissues, including the heart, suggests an increase in membrane permeability, cardiomyocyte injury and cell death. Administration of CUR + PIP to HFD-fed rats markedly influenced cardiac marker levels, suggesting a potential protective effect against obesity-induced myocardial damage.

In the present study, the assessment of CK-MB activity in cardiac damage is consistent with some previous reports but differs from others. tissue homogenates revealed a significant reduction in the HFD group compared with the control, confirming myocardial membrane damage and enzyme leakage into the circulation. Treatment with CUR + PIP markedly restored CK-MB levels toward normal values, indicating improved cardiomyocyte membrane integrity and supporting the biochemical findings observed in serum. These results are in line with Welsh et al. (2002) ^3^ reporting that curcumin exerts cardioprotective effects through the stabilization of sarcolemmal membranes and inhibition of lipid peroxidation within cardiac tissue.

Moreover, gene expression analysis demonstrated that NF-κB was markedly upregulated in HFD-fed rats, indicating activation of inflammatory signaling pathways associated with obesity-induced cardiac injury. Treatment with CUR + PIP significantly downregulated NF-κB expression, suggesting an attenuation of inflammatory signaling.

The underlying mechanism may involve modulation of oxidative stress, as obesity-induced overproduction of reactive oxygen species (ROS) can activate NF-κB and stimulate the release of pro-inflammatory cytokines such as TNF-α and IL-6. Curcumin is thought to inhibit this cascade by reducing ROS generation and preventing NF-κB nuclear translocation, while piperine may potentiate this effect by improving curcumin bioavailability. These findings align with previous reports indicating that the CUR + PIP combination exerts anti-inflammatory and cardioprotective effects through inhibition of NF-κB signaling ^66,69,84^.

Histopathological examination of the hearts from the present study of HFD rats revealed degeneration of myofibers at the tissue level, infiltration of inflammatory cells, congestion of blood vessels, and pyknosis of nuclei, accompanied by significant enlargement of interstitial space. These results agree with those of Feriani et al.^61^ and Basheer et al.^88^. Furthermore, administration of CUR + PIP to HFD rats may improve cardiac tissue, increase myofiber size, significantly reduce the interstitial space, and increase the presence of central nuclei. The results of the studies of Divakar et al.^89^ and Mohamed^90^were also similar.

## Conclusion

In summary, curcumin combined with piperine (CUR + PIP) exhibited significant cardioprotective effects against obesity-induced cardiac toxicity through multiple interrelated mechanisms. The treatment attenuated oxidative stress and inflammatory responses, improved antioxidant defense, and maintained ionic homeostasis, leading to the preservation of myocardial integrity. These improvements were supported by both biochemical and histopathological findings, indicating a remarkable recovery of cardiac structure and function.

Collectively, these results suggest that CUR + PIP exerts a multi-mechanistic protective role against obesity-related cardiac alterations, primarily through its antioxidant, anti-inflammatory, and membrane-stabilizing activities. Further investigations are required to elucidate additional molecular pathways involved and to evaluate their translational potential in human studies.

In summary, CUR + PIP therapy demonstrated significant protective effects against obesity-related cardiotoxicity through multiple mechanisms, including the reduction of inflammation, oxidative stress, and histological damage, as well as the enhancement of antioxidant capacity. These findings indicate that CUR + PIP may have beneficial effects in mitigating obesity-related cardiac alterations in rats, and further studies are needed to explore their potential therapeutic role in humans.

## Data Availability

The datasets generated and analyzed during the current study are available from the corresponding author on reasonable request.

## Abbreviations

HFD: High-fat diet
CUR: Curcumin
PIP: Piperine
i.p.: Intraperitoneal
W/V: Weight/Volume
˚C: Degree celsius
TC: Total cholesterol
TGs: Triglycerides
HDL-C: High-density lipoprotein cholesterol
LDL: Low-density lipoprotein cholesterol
CRI-I: Castelliʼs Risk Index I
CRI-II: Castelliʼs Risk Index II
AC: Atherogenic Coefficient
Gpx: Glutathione peroxidase
SOD: Superoxide dismutase
CAT: Catalase
MDA: Malondialdehyde
NO: Nitric oxide
ROS: Reactive oxygen species
Na+/K+-: ATPase Sodium/ Potassium ATPase
TNF-α: Tumor necrosis factor-α
IL-1: Interleukin1
IL-6: Interleukin6
CRP: C-reactive protein
MCP-1: Monocyte Chemoattractant Protein-1
PAI-1: Plasminogen Activator Inhibitor 1
CK-MB: Creatine Kinase-MB
Na+: Sodium (Na+)
K+: Potassium (K+)
CL−: Chloride (Cl−)
Ca2: Calcium (Ca2+)
H&E: Haematoxylin and Eosin
